# Strict adherence to malaria rapid test results might lead to a neglect of other dangerous diseases: a cost benefit analysis from Burkina Faso

**DOI:** 10.1186/1475-2875-10-226

**Published:** 2011-08-04

**Authors:** Zeno Bisoffi, Sodiomon B Sirima, Filip Meheus, Claudia Lodesani, Federico Gobbi, Andrea Angheben, Halidou Tinto, Bouma Neya, Klara Van den Ende, Annalisa Romeo, Jef Van den Ende

**Affiliations:** 1 Centre for Tropical Diseases, S. Cuore Hospital, 37024 Negrar (Verona), Italy; 2 Projet AnKaHeresso, BP 292 Bobo Dioulasso, Burkina Faso; 3 Centre National de Recherche et de Formation sur le Paludisme, Ministry of Health, B.P. 2208, Ouagadougou 01, Burkina Faso; 4 Department of Public Health, Prince Leopold Institute of Tropical Medicine, Nationalestraat 155, Antwerp, Belgium; 5 Médecins sans Frontières, via Volturno 58, 00185 Rome, Italy; 6 Centre Muraz, BP 390, Bobo Dioulasso 01, Burkina Faso; 7 Department of Clinical Sciences, Prince Leopold Institute of Tropical Medicine, Nationalestraat 155, Antwerp, Belgium

## Abstract

**Background:**

Malaria rapid diagnostic tests (RDTs) have generally been found reliable and cost-effective. In Burkina Faso, the adherence of prescribers to the negative test result was found to be poor. Moreover, the test accuracy for malaria-attributable fever (MAF) is not the same as for malaria infection. This paper aims at determining the costs and benefits of two competing strategies for the management of MAF: presumptive treatment for all or use of RDTs.

**Methods:**

A cost benefit analysis was carried out using a decision tree, based on data previously obtained, including a randomized controlled trial (RCT) recruiting 852 febrile patients during the dry season and 1,317 in the rainy season. Cost and benefit were calculated using both the real adherence found by the RCT and assuming an ideal adherence of 90% with the negative result. The main parameters were submitted to sensitivity analysis.

**Results and discussion:**

At real adherence, the test-based strategy was dominated. Assuming ideal adherence, at the value of 525 € for a death averted, the total cost of managing 1,000 febrile children was 1,747 vs. 1,862 € in the dry season and 1,372 vs. 2,138 in the rainy season for the presumptive vs. the test-based strategy. For adults it was 2,728 vs. 1,983 and 2,604 vs. 2,225, respectively. At the subsidized policy adopted locally, assuming ideal adherence, the RDT would be the winning strategy for adults in both seasons and for children in the dry season.

At sensitivity analysis, the factors most influencing the choice of the better strategy were the value assigned to a death averted and the proportion of potentially severe NMFI treated with antibiotics in patients with false positive RDT results. The test-based strategy appears advantageous for adults if a satisfactory adherence could be achieved. For children the presumptive strategy remains the best choice for a wide range of scenarios.

**Conclusions:**

For RDTs to be preferred, a positive result should not influence the decision to treat a potentially severe NMFI with antibiotics. In the rainy season the presumptive strategy always remains the better choice for children.

## Background

Several economic studies have been carried out on malaria management with rapid diagnostic tests (RDTs) as a guide to initiate treatment, compared with the previously common presumptive approach and/or with microscopy [1-9]. RDTs have generally been found to be accurate, reliable and cost effective. New WHO guidelines on malaria management [10] state that a laboratory diagnosis of malaria is required for treatment, in all endemic settings and for all age groups including (contrarily to previous guidelines) [11] children under five. A thorough, multi phase evaluation of several commercially available RDTs is currently underway, and the results of the first two phases [12,13] show that many of these tests are highly sensitive down to a very low parasite density of 200 parasites/μL and also highly specific. Recently, an estimate of RDT accuracy for malaria-attributable fever rather than for simple infection in rural Burkina Faso showed that the test under study (Paracheck Device^®^, Orchid Biomedical Systems, Goa, India) had an excellent sensitivity, but a poor specificity [14]. Moreover, in the same region, the adherence of prescribers to the negative test result was lower than 20% for both adults and children [15]. An excessive reliance on positive results is not without consequences, either, as a false positive test may suggest refraining from treatment with antibiotics: this was indeed the case of one of the children in the RCT who subsequently died, presumably of pneumonia [14].

The aim of this study was to estimate the costs and benefits of a strategy of presumptive management of malaria-attributable fever compared to RDT based management in a rural setting in Burkina Faso. Various scenarios were developed incorporating both the observed (low) adherence to the test results as well as an "improved" adherence that can be obtained through better training and motivation of health personnel. Moreover, in the current algorithm used in the study area, the positive result is followed by malaria treatment with no other options, while only the negative result may lead to a further diagnostic workup for other diseases. The consequences of not providing antibiotic treatment to patients who may need it following a false positive test result were then assessed.

### Study design

The data for this study is derived from a randomized controlled trial carried out in Burkina Faso on the use of a malaria rapid diagnostic test (RDT) during the dry season (852 febrile patients) and the rainy season (1,317 febrile patients) [15] and from a previous assessment of the RDT accuracy for malaria-attributable fever [14]. When primary data was not available or too limited, assumptions were used based on criteria outlined in the following sections. The outcome of each strategy is defined by the following parameters with different values for adults (Table 1) and children (Table 2): the prevalence of malaria-attributable fever in adults and children in both seasons; the RDT sensitivity and specificity for malaria-attributable fever in both seasons; the proportion of febrile patients (with and without malaria) treated with anti-malarials and with antibiotics, in the presumptive arm and in the RDT arm (according to the test result), in both seasons; and the excess mortality (after subtracting the proportion of deaths that would occur in correctly treated patients) from untreated malaria as well as from untreated bacterial non malarial febrile illness (NMFI).

**Table 1:** Parameters used in the study, children < 5 years

EPIDEMIOLOGICAL PARAMETERS, CHILDREN		**Source**^**1**^
Malaria attributable fevers (MAF)/febrile patients (dry season)	3.18%	Primary
Malaria attributable fevers (MAF)/febrile patients (rainy season)	63.05%	Primary
Malaria parasite density > 40,000/μL (both seasons)	37.6%	Primary
Malaria parasite density ≤40,000/μL (both seasons)	62.4%	Primary
Death rate treated MAF, high parasite density	0.60%	Primary
Excess death rate untreated MAF, high parasite density	9.4%	Assumption
Excess death rate untreated MAF low parasite density	0.25%	Assumption
Excess death rate untreated MAF in RDT neg	0.1%	Assumption
Death rate treated NMFI	0.84%	Primary
Proportion of potentially fatal non malarial fever (PFNM)	20%	Assumption
Excess death rate untreated PFNM	7%	Assumption
**CASE MANAGEMENT, PRESUMPTIVE BRANCH, CHILDREN**		
Anti-malarial treatment among MAF	94.1%	Primary
Anti-malarial treatment among MAF, high parasite density	100%	Primary
Anti-malarial treatment among MAF, low parasite density	87%	Assumption
Anti-malarial treatment among those treated with antibiotics	86.3%	Primary
Anti-malarial treatment among those not treated with antibiotics	97.4%	Primary
Antibiotic treatment among NMFI	64.5%	Primary
Antibiotic treatment among PFMN	90%	Assumption
Antibiotic treatment among patients presumptively treated for malaria	54.4%	Primary
Antibiotic treatment among PFMN presumptively treated for malaria	76%	Assumption
Antibiotic treatment among patients not presumptively treated for malaria	89.7%	Primary
Antibiotic treatment among PFMN not treated for malaria	100%	Assumption
**CASE MANAGEMENT, RDT, CHILDREN**		
Anti-malarial treatment among RDT+, high parasite density (hpd)	100%	Primary
Anti-malarial treatment among RDT+, low parasite density (lpd)	98.1%	Primary
Anti-malarial treatment among RDT-	10.0%	Assumption^2^
Antibiotic treatment among RDT+	52.9%	Primary
Antibiotic treatment among PFNM RDT+	76%	Assumption
Antibiotic treatment among RDT-	86.1%	Assumption^2^
Antibiotic treatment among PFNM RDT-	98%	Assumption
**RDT ACCURACY, CHILDREN**		
RDT sensitivity, malaria attributable fever (MAF), lpd, dry season	95%	Primary
RDT specificity, malaria attributable fever (MAF), dry season	71%	Primary
RDT sensitivity, malaria attributable fever (MAF), lpd, rainy season	95.9%	Primary
RDT specificity, malaria attributable fever (MAF), rainy season	36.7%	Primary
RDT sensitivity, MAF, high parasite density	100%	Primary
**COSTS, CHILDREN**		
Cost of RDT	0.71	Ref. 26
Cost of anti-malarial treatment, Coartem (average, €)	1	Ref. 26
Cost of antibiotic treatment (average, €)	0.5	Estimate
Life Value (€) corresponding to 25 US $*YLL	525	(see text)
Life Value (€) corresponding to 150 US $*YLL	3150	(see text)

^1 ^Primary data obtained from previous RCT (Ref. 15) and from previous assessment of the RDT accuracy (Ref. 14); assumptions based on estimates from primary data, expert opinion and previous literature (see explanation in text).

^2 ^Assuming "ideal" 90% adherence to the negative test result (see explanation in text).

**Table 2:** Parameters used in the study, children ≥ 5 years and adults

EPIDEMIOLOGICAL PARAMETERS		**Source**^**1**^
Malaria attributable fevers (MAF)/febrile patients (dry season)	1.7%	Primary
Malaria attributable fevers (MAF)/febrile patients (rainy season)	25.1%	Primary
Malaria parasite density > 40,000/μL (both seasons)	36.8%	Primary
Death rate treated MAF, high parasite density	0.0%	Primary
Excess death rate untreated MAF, high parasite density	0.4%	Assumption
Excess death rate untreated MAF low parasite density	0.0%	Assumption
Excess death rate untreated MAF in RDT neg	0.0%	Assumption
Death rate treated NMFI	0.84%	Primary
Proportion of potentially fatal non malarial fever (PFNM)	20%	Assumption
Excess death rate untreated PFNM	7%	Assumption
**CASE MANAGEMENT, PRESUMPTIVE BRANCH, ADULTS**		
Anti-malarial treatment among MAF	97%	Primary
Anti-malarial treatment among MAF, high parasite density	97.3%	Primary
Anti-malarial treatment among MAF, low parasite density	97%	Assumption
Anti-malarial treatment among those treated with antibiotics	79.3%	Primary
Anti-malarial treatment among those not treated with antibiotics	94.8%	Primary
Antibiotic treatment among NMFI	56.1%	Primary
Antibiotic treatment among PFMN	90%	Assumption
Antibiotic treatment among patients presumptively treated for malaria	47.1%	Primary
Antibiotic treatment among PFMN presumptively treated for malaria	86%	Assumption
Antibiotic treatment among patients not presumptively treated for malaria	80.8%	Primary
Antibiotic treatment among PFMN not treated for malaria	99%	Assumption
**CASE MANAGEMENT, RDT, ADULTS**		
Anti-malarial treatment among RDT+, high parasite density (hpd)	100%	Primary
Anti-malarial treatment among RDT+, low parasite density (lpd)	96.4%	Primary
Anti-malarial treatment among RDT-	10%	Assumption
Antibiotic treatment among RDT+	42.5%	Primary
Antibiotic treatment among PFNM RDT+	86%	Assumption
Antibiotic treatment among RDT-	77.4%	Assumption
Antibiotic treatment among PFNM RDT-	98.6%	Assumption
**RDT ACCURACY, ADULTS**		
RDT sensitivity, malaria attributable fever (MAF), lpd, dry season	91.6%	Primary
RDT specificity, malaria attributable fever (MAF), dry season	78.7%	Primary
RDT sensitivity, malaria attributable fever (MAF), lpd, rainy season	95.3%	Primary
RDT specificity, malaria attributable fever (MAF), rainy season	77.4%	Primary
RDT sensitivity, MAF, high parasite density	99%	Primary
**COSTS, ADULTS**		
Cost of RDT	0.71	(ref. 26)
Cost of anti-malarial treatment, Coartem (average, €)	2	(ref. 26)
Cost of antibiotic treatment (average, €)	0.4	Estimate
Life Value (€) corresponding to 25 US $*YLL	525	(see text)
Life Value (€) corresponding to 150 US $*YLL	3150	(see text)

^1 ^Primary data obtained from previous RCT (ref. 15) and from previous assessment of the RDT accuracy (ref. 14); assumptions based on estimates from primary data, expert opinion and previous literature (see explanation in text)

The prevalence of malaria-attributable fever was taken rather than infection as a proxy of clinical malaria since it allows for a better classification of cases and related outcomes. When using data on infection, outcomes (including death) could be falsely attributed to malaria instead of NMFI (or vice versa) and thereby lead to biased conclusions. The specificity of RDTs was not the same in the two seasons nor in the two age groups, while sensitivity was related to parasite density. Therefore, age, season and parasite density-specific data were used.

Probabilities related to mortality were derived from the randomized controlled trial [15]. When data was not or partially available, the following assumptions were used: for malaria, as the vast majority of patients in the trial were treated, no death was observed in untreated patients. Only one death was reported in a treated child, the latter corresponding to 0.25% case fatality rate of treated, clinical malaria, or 0.6% if the denominator is malaria with higher parasite density (> 40,000/μL or >1%). The case fatality rate (CFR) of untreated malaria with higher parasite density in children < 5 years was assumed to be about 10%, based on expert opinion. For lower parasite densities a lower CFR was assumed (Table 1). The overall CFR of untreated malaria (any parasite density) in children was estimated to be about 2.5%, which is comparable with previous estimates in other African countries [1,16,17]. Nevertheless, almost all the untreated malaria cases were treated with antibiotics, and the drug used in most cases was cotrimoxazole, which is still partly effective on malaria in the area. An estimate was then obtained of malaria CFR in cases not treated with an anti-malarial, adjusting for the proportion treated with cotrimoxazole. The CFR for untreated malaria in RDT negative children was assumed to be much lower (0.1%), taking into account the lower parasite density and the favourable outcome of such patients observed in other studies [18-20] as well as in a previous study on cases missed by microscopy [21]. For adults, all the estimates were obviously much lower (Table 2). As for non-malarial febrile illness (NMFI), data from the trial showed that the CFR in patients treated with antibiotics approached 1% both in adults and children. No death from NMFI was observed in patients not treated with antibiotics in the presumptive arm in either age group, and only one in the RDT arm (a child with a probable pneumonia who was not treated with antibiotics after a false positive RDT in the dry season). The only possible explanation of this apparent paradox is that clinical officers tend to correctly estimate the potential severity of a febrile illness and, therefore, treat most of the potentially fatal non-malarial fevers, while treating a lower proportion of mild NMFI. Therefore, primary data on antibiotic treatment of NMFI obtained from the trial were adjusted to assume a higher proportion of antibiotic treatment for potentially severe NMFI (assumed to account for about 20% of all NMFI) and a correspondingly lower proportion for milder NMFI. A fatality rate of 10% was assumed for untreated cases of potentially severe NMFI, corresponding to a death rate of 2% over all untreated NMFI. These assumptions were based on previous estimates and on expert opinion and were able to predict the real figures found by the trial. Figures used for the analysis were those on excess mortality.

**Figure 1:**
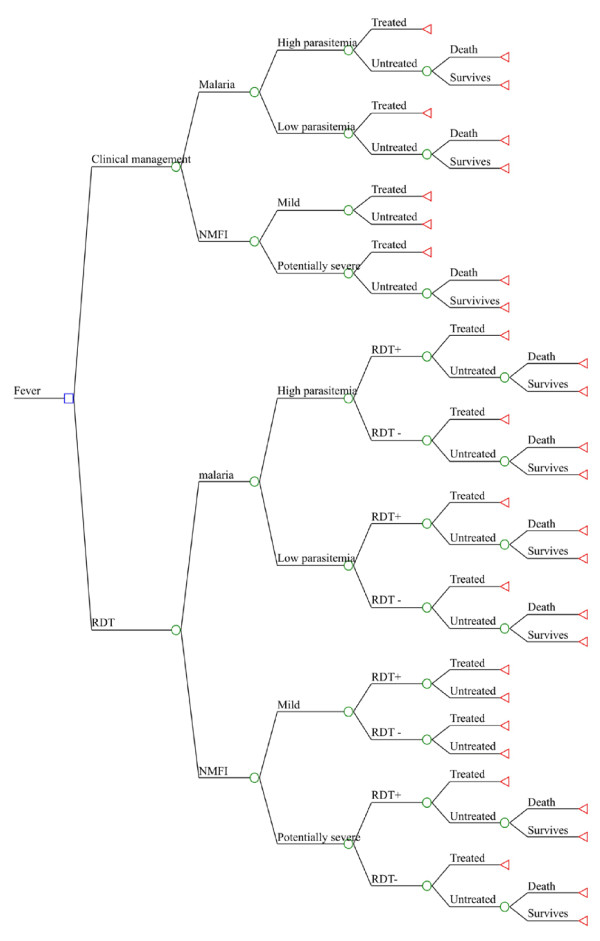
**Structure of the Decision Tree**.

The basis of the model is a decision tree comparing fever management on a presumptive basis versus management guided by a rapid diagnostic test for the detection of malaria for a hypothetical cohort of 1,000 patients presenting with axillary temperature ≥ 37.5 °C at a primary health centre (Figure 1). In this study we use cost-benefit analysis whereby the cost of each strategy is combined with its outcome expressed in monetary terms, in this case the value of deaths averted. Using the same decision tree structure, we estimated the outcome for four different groups: (i) children in the dry season, (ii) adults in the dry season, (iii) children in the rainy season and (iv) adults in the rainy season. Children were defined as patients below the age of five (<5yrs), and adults defined as patients of 5 years or more (≥5 yrs). For each cohort, two scenarios were modelled that differed in terms of the clinicians' adherence to the test results. First, the analysis was carried out using adherence rates as observed in the RCT [15]. The clinicians' adherence to the negative RTD result was very poor in both age groups and in both seasons. Interestingly, the adherence to the presumptive strategy for malaria treatment was not 100% but about 90% and also the adherence to the positive RDT result, although very high, was less than 100%. Nevertheless the proportion of cases treated increased with parasite density in both arms and approached 100% for parasite densities over 40,000/μL indicating that, similarly to NMFI (see above), potentially severe cases are more likely to be treated. The proportion of patients treated with antibiotics was also much higher in patients not treated for malaria, and vice versa (Tables 1 and 2). A further analysis was performed assuming that through better training, motivation and supervision it would be possible to substantially increase the adherence to the negative test result, consistently with findings from other studies: [18,22,23] an ideal value of 90% was assumed, compared to less than 20% observed in the field. The adherence to the positive test was assumed to be 100%, while the adherence rates of the presumptive management of febrile patients were not varied.

The cost benefit approach implies that a monetary value be assigned to the main health outcome, that is, a death averted by correct case management by either strategy. We started the analysis with the very low value of 525 € (euro) for a death averted, which roughly corresponds to 25 US $ for a YLL avoided (that is a suggested benchmark for a "highly attractive" health intervention) [1]. The upper range in the tree was 3,150 € (euro), which roughly corresponds to 150 US $ for a YLL, a benchmark for an attractive intervention [1]. For the estimate of years of life lost (YLL) we used the equation described by Fox-Rushby and Hanson [24]. In Burkina Faso, according to the most recent estimates [25], the life expectancy at birth was 52.9 years. The median age of the study cohort was one year for children and 18 years, for adults. A death at this age would correspond to YLLs[0.03,1,0.04] = 30.8 for a child and = 30.4 for an adult. For convenience, both were set at 30.

Costs were considered from a societal perspective and included the cost of the rapid diagnostic test, malaria drug treatment cost and the cost of antibiotic treatment for children and adults. Prices for both the anti-malarials (artemisinin-based combination therapy (ACT); Coartem^®^) and the RDT (Paracheck Device^®^, Orchid Biomedical Systems, Goa, India) were obtained from a leading international no-for-profit supplier (2010 price list) [26]. These costs are currently partly subsidized by external sources. In the study area, the RDT is currently fully subsidized and performed at no charge and the ACT average subsidized cost is 100 CFA (0.15 €) for children under 5 and 250 CFA (0.38 €) for adults (see Tables 1 and 2). The analysis was first performed considering all inputs at their full cost and subsequently using the subsidized costs.

Costs in local currency (CFA franc) were converted to Euros at a (fixed) exchange rate (100 CFA francs = 1 former French franc = € 0.00152449). The value of YLLs expressed in US dollars was converted to Euros at US$1.43/euro (January 21^st^, 2010). Analyses were performed using TreeAge Pro Suite v1.0.2 (Treeage Inc, Williamstown, MA, USA).

### Sensitivity analysis

As a previous, similar study [1] showed that results were most sensitive to the monetary value assigned to YLLs, the threshold life value (that is the monetary value assigned to a death averted at which the two strategies become equivalent) was first calculated for each group/season, when applicable.

The compliance as well as the other main factors were also submitted to a sensitivity analysis. The range of variation for primary data was provided by the 95% confidence interval, while that for assumptions was wide enough to challenge the robustness of the main conclusions. The hypothesis that a false positive RDT result (for malaria-attributable fever) might influence the management of NMFI was also tested. For the main analysis the results of the primary data were used, i.e. the proportion of NMFI with a false positive RDT treated with antibiotics was the same as among patients presumptively treated for malaria. For sensitivity analysis, a higher proportion of patients was assumed to be treated only with an anti-malarial without antibiotics in case of a positive RDT result, if compared with the presumptive management.

## Results

### Patient population

The characteristics of the study population are described in detail elsewhere [14,15]. To summarize, primary data for this study were obtained from 852 febrile patients in the dry season and 1,317 in the rainy season. The proportion of children under five (median age: 1 year) and adults (median age 18 years) was 50.9% and 49,1% respectively. The main data on malaria and NMFI prevalence and on case management are resumed in Table 1 (children) and Table 2 (adults).

### Cost benefit at observed versus ideal adherence

At the adherence levels found by the trial, the test-based strategy was clearly disadvantageous in both seasons and for both age groups. In particular, total costs of the presumptive strategy compared to the test-based strategy for children were 1,747€ vs. 2,615€ in the dry season and 1,372€ vs. 2,284 € in the rainy season. For adults total costs were 2,728€ vs. 3,369€ and 2,604€ vs. 3,271€ respectively (Table 3).

**Table 3:** Direct cost (test and treatment cost) and comprehensive cost (including estimate of life value) of the presumptive versus the test based strategy at "real life" adherence levels to both strategies (see text): test and treatment cost not subsidized

Variables	Children, dry season	Children, rainy season	Adults, dry season	Adults, rainy season
Direct cost, presumptive strategy	**1032**	**1033**	**2005**	**2048**
Direct cost, test-based strategy	1713	1767	2699	2758
Comprehensive cost, presumptive strategy	**1747**	**1372**	**2728**	**2604**
Comprehensive cost, test-based strategy	2615	2284	3369	3271
Threshold life value*	n.a. (test dominated)	n.a. (test dominated)	6958	9071

Costs expressed in € (euro) for 1000 patients managed with either strategy, assuming a life value of 525 € **(better option in bold)**

* Life value at which the two strategies become equivalent at sensitivity analysis

Assuming an ideal adherence of 90% by health providers to the negative test results total costs were 1,747€ vs. 1,862 € in the dry season and 1,372€ vs. 2,138€ in the rainy season for the presumptive vs. the test based strategy, while for adults it was 2,728€ vs. 1,983€ and 2,604€ vs. 2,225€ respectively (Table 4).

**Table 4:** Direct cost (test and treatment cost) and comprehensive cost (including estimate of life value) of the presumptive versus the test based strategy at "ideal" adherence levels to both strategies (see text): test and treatment cost not subsidized

Variables	Children, dry season	Children, rainy season	Adults, dry season	Adults, rainy season
Direct cost, presumptive strategy	**1032**	**1033**	2005	2047
Direct cost, test-based strategy	1242	1684	**1685**	**1988**
Comprehensive cost, presumptive strategy	**1747**	**1372**	2728	2604
Comprehensive cost, test-based strategy	1862	2138	**1983**	**2225**
Threshold life value*	1151	n.a. (test dominated)	n.a. (pres. dominated)	n.a. (pres. dominated)

Costs expressed in € (euro) for 1000 patients managed with either strategy, assuming a life value of 525 € **(better option in bold)**

* Life value at which the two strategies become equivalent at sensitivity analysis

### Cost benefit at current subsidized costs

When the subsidized costs for anti-malarial drugs and rapid diagnostic tests are considered (i.e. the financial cost to the malaria control programme and patients) rather than the full cost (that includes donor support) at current adherence levels, the presumptive strategy is still preferred for children in both seasons while the test based strategy is the more attractive for adults (Table 5). At ideal adherence levels, the presumptive strategy is dominated for adults in both seasons and for children in the dry season, but still remains the better alternative for children in the rainy season (Table 6).

**Table 5:** Direct cost (test and treatment cost) and comprehensive cost (including estimate of life value) of the presumptive versus the test based strategy at the current "real life" adherence levels to both strategies: test and treatment cost subsidized (see text)

Variables	Children, dry season	Children, rainy season	Adults, dry season	Adults, rainy season
Direct cost, presumptive strategy	264	257	**608**	**608**
Direct cost, test-based strategy	**256**	**255**	609	610
Comprehensive cost, presumptive strategy	**979**	**596**	1330	1164
Comprehensive cost, test-based strategy	1157	760	**1279**	**1125**
Threshold life value*	23	7	14	25

Costs expressed in € (euro) for 1000 patients managed with either strategy, assuming a life value of 525 € **(better option in bold)**

* Life value at which the two strategies become equivalent at sensitivity analysis

**Table 6:** Direct cost (test and treatment cost) and comprehensive cost (including estimate of life value) of the presumptive versus the test based strategy at "ideal" adherence levels to both strategies: test and treatment cost subsidized (see text)

Variables	Children, dry season	Children, rainy season	Adults, dry season	Adults, rainy season
Direct cost, presumptive strategy	264	257	608	608
Direct cost, test-based strategy	**209**	**247**	**485**	**516**
Comprehensive cost, presumptive strategy	978	**596**	1330	1164
Comprehensive cost, test-based strategy	**828**	697	**782**	**752**
Threshold life value*	n.a. (pres. dominated)	44	n.a. (pres. dominated)	n.a. (pres. dominated)

Costs expressed in € (euro) for 1000 patients managed with either strategy, assuming a life value of 525 € **(better option in bold)**

* Life value at which the two strategies become equivalent at sensitivity analysis

### Sensitivity analysis

The sensitivity analysis showed that, besides the adherence and the costs of the test and treatment, the results were influenced by the monetary value assigned to a death averted and by the potential effect of the RDT (false) positive result on the treatment of NMFI with antibiotics.

The effect of the life value on the main results, "ceteris paribus", is resumed in Tables 3 to 6 by the "threshold life value", that is the monetary value assigned to a death averted at which the two strategies are equivalent. At the real adherence level found by the trial, for children in both seasons the test-based strategy was dominated for any value assigned to a life saved, while for adults in the dry and in the rainy season the test based strategy would only become advantageous at a life value > 6,958€ and 9,071 € (Table 3), corresponding to about 232 and 302 € per YLL prevented, respectively.

At the "ideal" adherence level, for adults in both seasons the presumptive strategy was dominated for any value assigned. For children, in the dry season, the test-based strategy became advantageous at a life value of > 1,151€, corresponding to a YLL value of 38 €, while in the rainy season the test based strategy was dominated (Table 4). Given the current subsidized costs, the test-based strategy is the winning option for adults, regardless the adherence, while for children the same strategy becomes advantageous at the ideal adherence level, but only in the dry season (Tables 5 and 6).

### Effect of the proportion of RDT false positive NMFI treated with antibiotics

**Figure 2:**
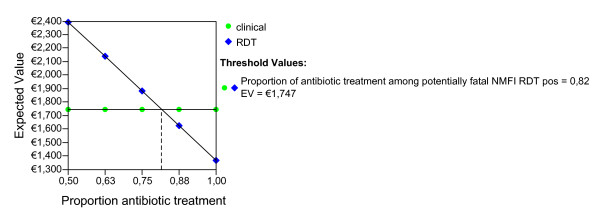
**Children, dry season**. Sensitivity Analysis on Proportion of antibiotic treatment among potentially fatal NMFI RDT pos.

**Figure 3:**
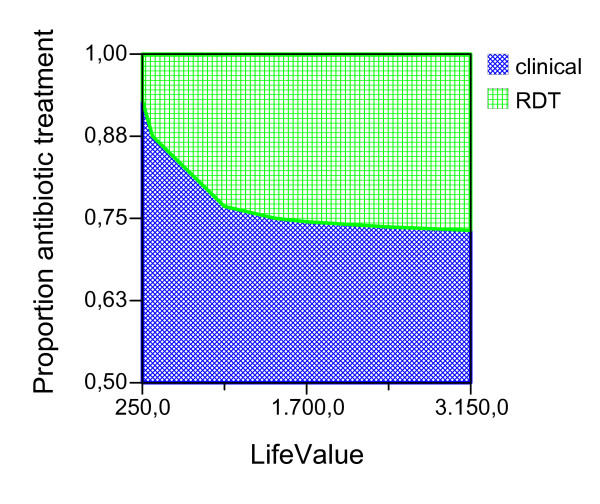
**Children, dry season**. Sensitivity Analysis on Life Value and Proportion of antibiotic treatment among potentially fatal NMFI RDT pos.

**Figure 4:**
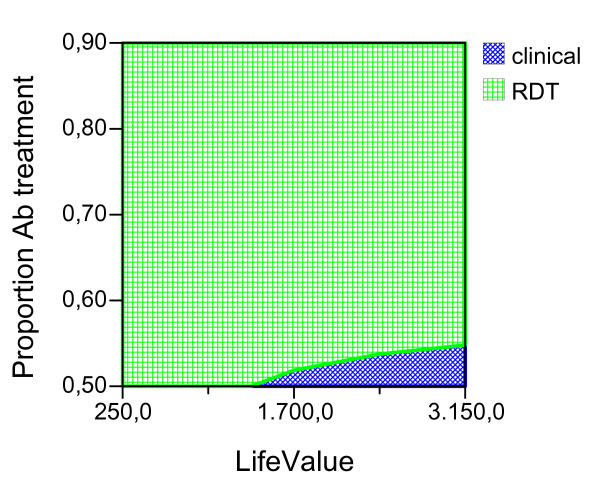
**Adults, dry season**. Sensitivity Analysis on Life Value and Proportion of antibiotic treatment among potentially fatal NMFI RDT pos.

**Figure 5:**
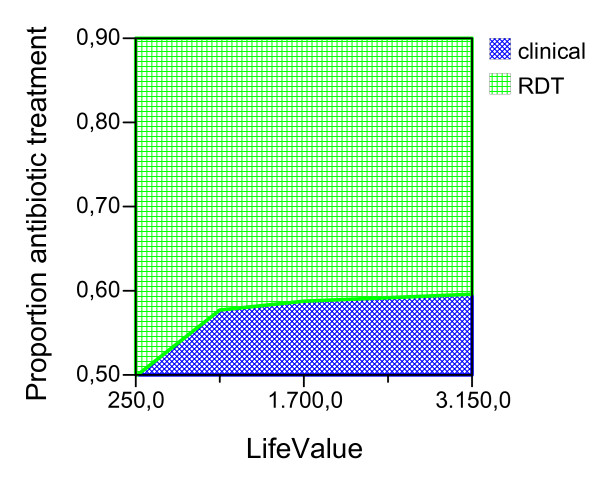
**Adults, rainy season**. Sensitivity Analysis on Life Value and Proportion of antibiotic treatment among potentially fatal NMFI RDT pos.

**Figure 6:**
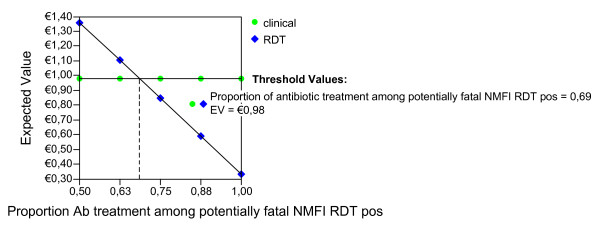
**Children, dry season, subsidized policy**. Sensitivity Analysis on Proportion of antibiotic treatment among potentially fatal NMFI RDT pos.

**Figure 7:**
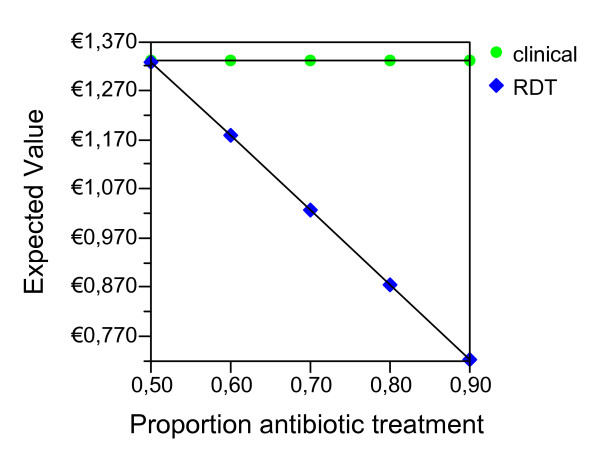
**Adults, dry season, subsidized policy**. Sensitivity Analysis on Proportion of antibiotic treatment among potentially fatal NMFI RDT pos.

**Figure 8:**
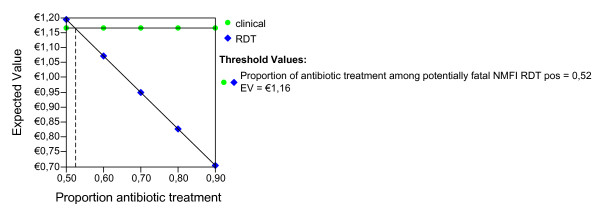
**Adults, rainy season, subsidized policy**. Sensitivity Analysis on Proportion of antibiotic treatment among potentially fatal NMFI RDT pos.

**Figure 9:**
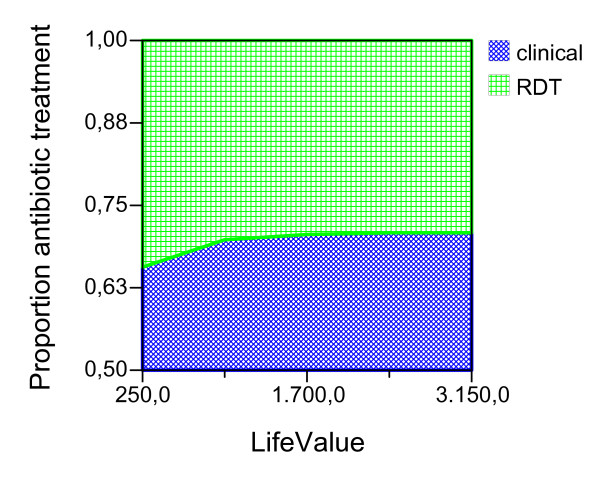
**Children, dry season, subsidized policy**. Sensitivity Analysis on Life Value and Proportion of antibiotic treatment among potentially fatal NMFI RDT pos.

**Figure 10:**
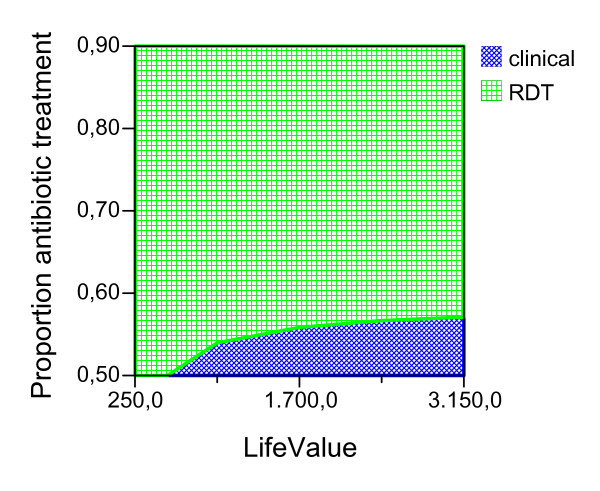
**Adults, dry season, subsidized policy**. Sensitivity Analysis on Life Value and Proportion of antibiotic treatment among potentially fatal NMFI RDT pos.

**Figure 11:**
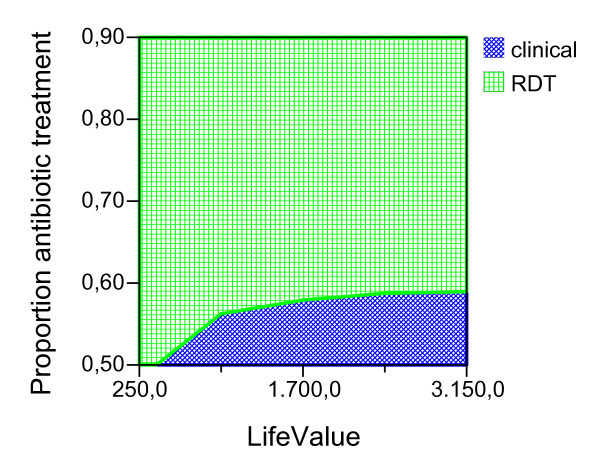
**Adults, rainy season, subsidized policy**. Sensitivity Analysis on Life Value and Proportion of antibiotic treatment among potentially fatal NMFI RDT pos.

Given the ideal adherence values of the main analysis, assuming that the improved confidence with the test result would have the effect of decreasing the proportion of antibiotic treatment in NMFI with a false positive RDT, we report the possible effect of this variable for both seasons and age groups (Figures 2, 3, 4, 5, 6, 7, 8, 9, 10, 11). For children in the dry season the RDT based strategy would only become advantageous if the proportion of potentially fatal NMFI treated with antibiotics among RDT positives were higher than 82% (Figure 2). In a two-way sensitivity analysis including life value (Figure 3), the required proportion of antibiotic treatment would remain over 75% even for much higher values assigned to life. For children in the rainy season, the test-based strategy would remain dominated for any value assigned to both variables. For adults in both seasons, the RDT based strategy would remain the preferred one if more than half potentially fatal NMFI with a positive test result were treated with antibiotics (Figures 4 and 5). Even at the current subsidized policy (see above), the test-based strategy would be no longer advantageous if the proportion of potentially fatal NMFI treated with antibiotics among RDT (false) positives dropped to < 69%, < 50% and < 52% for children in the dry season and adults in the dry and rainy season, respectively (Figures 6 to 8). The two-way sensitivity analysis including the life value (Figures 9 to 11) gave very similar results.

## Discussion

### General findings

In a hyperendemic malaria region, with a very marked difference in transmission intensity such as in the study area, the optimal choice in terms of cost benefit between the two competitive strategies is not straightforward. At the adherence level (to the negative test result) found by the trial in the study area [15], a test-based policy is clearly disadvantageous for both seasons and age groups, confirming that the prescribing behaviour is a crucial operational factor affecting the new policy recommended by the WHO [10].

Assuming that through better training and motivation care providers become more confident on the negative test result using an ideal adherence of 90%, the test-based strategy would become the best option for adults in both seasons, but not for children. At the ideal adherence and leaving other factors unchanged, a test-based policy would dominate a presumptive strategy at the current subsidized pricing policy, with the notable exception of children in the rainy season.

Besides adherence and cost of the test and treatment, the sensitivity analysis pointed to the value of life and to the effect of false positive RDT results on antibiotic prescription as key determinants. Given the "ideal" adherence, at a slightly higher life value the test-based strategy would become attractive for children, but only in the dry season. No increase in life value would make the RDT based strategy attractive for children in the rainy season, while a decrease in the proportion of potentially fatal NMFI treated with antibiotics following a false positive RDT would radically change the main conclusions and make the test based policy the less attractive also for the other three groups.

### Comparison with previous studies

#### Methodology

This analysis differs from previous studies in two main methodological aspects. First, the RDT accuracy values used for the analysis refer to malaria-attributable fever, and not simply to malaria infection [14]. Though the accuracy values given are based on estimates, the advantage is a more correct classification of malaria and of non-malarial febrile illness (NMFI). Secondly, data on mortality, both for malaria and NMFI, are not simply derived from general literature data and/or from expert opinion, but on estimates based on real data found by a previous trial, and so are all data on case management.

#### Adherence

The effect of adherence has already been discussed [1,27]. At a very low adherence level to the negative result a test based strategy cannot be cost effective: the test only adds an unnecessary cost, if its result will not change the decision to treat [27]. Nevertheless, in other study settings a much better compliance was observed suggesting that a similar result could be obtained in Burkina Faso too [18,22,28]. However, the results of this study indicate that even at an ideal adherence the test based strategy offers a clear and undisputable advantage for adults only.

#### Life value

Similarly to a study carried out with a similar methodology the results were most sensitive to the cost assigned to a year of life lost [1]. In this study the value assigned to a death averted was crucial to indicate the best option for some of the scenarios discussed, while for other the best choice was insensitive to this factor, obviously difficult to estimate, especially for low and very low income countries.

#### Antibiotic prescription

Previous studies suggest that clinicians and nurses tend to treat fewer patients with antibiotics when a malaria test is positive which is by no means surprising [9,29]. Nurses active in the study area when asked what the main advantage of the RDT were answered that "the positive result is useful as it permits to rule out other diseases" (Federico Gobbi, personal communication, November 2010). The results on the effect of antibiotic prescription after a false positive test are understandable, considering that the test specificity for malaria-attributable fever was particularly low. This is so, because a proportion of low-density infections that are detected by the RDT cannot be considered malaria in clinical terms and are, therefore, classified as false positive for this study purpose [14].

### Weaknesses and limitations

There are also a number of limitations to this study. Although most of the parameters used for the analysis are primary data obtained from direct observation or on best estimates from primary data, some of them are necessarily based on assumptions and/or expert opinion, which is common to most health economic studies. Given the weight of health outcome on the final result, when very small differences in outcome (deaths averted) are observed, the results need to be taken with extreme caution. This study did not consider certain costs such as material (disposable gloves, lancets) and labour cost that would have increased the cost of the test-based strategy. Time cost for patients and guardians and cost of referral and secondary care are not considered either because they are difficult to estimate and also because the previous trial showed that only a very negligible proportion of patients were referred [15]. Last but not least, this study assumes that only febrile patients are tested. Other studies found that care providers also tested a proportion of patients without fever or even without a recent fever history [19,20]. Should this practice become the rule, it would further hamper the potential cost saving of the test-based management.

### Possible impact

The previous trial did not show any significant difference in the proportion of antibiotic treatment between patients in the presumptive arm and those with a positive RDT result [15]. If an improved confidence in the test is obtained through better training and motivation, in order to achieve a better adherence to the negative result, it is only too logical to expect a corresponding, higher reliance on the positive result, as well, which would affect antibiotic prescription. It can be argued that the possible disastrous effect of a false positive RDT result on antibiotic prescription for potentially severe NMFI is compensated by a higher proportion of antibiotic prescription to RDT negatives. This is obviously true, but this effect is already accounted for in our model, as this proportion is estimated to be close to 100%.

In more general terms, this study shows that a test-based policy is advantageous only if it leads to improved medical decisions. The presumptive strategy certainly causes an over prescription of both anti-malarials and antibiotics, but the advantage is that most potentially severe cases are treated. RDTs should limit anti-malarial prescription through an "ideal" adherence to the negative test result [18,27]. The adverse consequences of a false negative RDT are probably negligible [18], though a word of caution is warranted for young children and infants [14], until definitive conclusions on safety are reached [30]. On the other hand, this study suggests that a positive RDT result should not necessarily become a reason to refrain from antibiotic treatment. Clinical guidelines, including the Integrated Management of Childhood Ilnesses (IMCI), should introduce RDTs at the right step. The treatment decision for NMFI should come before the RDT node in clinical algorithms, and the RDT result should be used as a guide for malaria treatment, but not to exclude other potential causes of fever.

Moreover, even under an "ideal" prescribing behaviour, the choice of a test-based strategy remains questionable for children under five, especially in the rainy season.

### Future research

Randomized controlled trials (RCTs) with adequate sample size on the outcome of a test-based versus a presumptive strategy are unfortunately lacking, which means that the widespread introduction of RDTs and their adoption as the optimal strategy everywhere and for all ages are not based on undisputable evidence [10]. If adequately powered RCTs are manageable and worth the necessary investment remains matter for discussion.

## Conclusions

For a test-based strategy to be preferred over a presumptive strategy in terms of cost and benefit, a near ideal adherence to the negative test result is needed, while a positive test result should not influence the decision to treat a potentially severe NMFI with antibiotics. Even if such ideal case management could be achieved, the RDT based strategy should be subsidized in order to be advantageous for young children in the dry season, while in the rainy season the presumptive strategy remains the better choice for children in all scenarios.

## Competing interests

The authors declare that they have no competing interests.

## Authors' contributions

ZB conceived the study design, wrote the study protocol, concurred to data analysis, wrote the draft and final version of the manuscript. SBS collaborated to the study design and writing of the study protocol. Contributed to draft versions, revised critically the manuscript. FM extensively revised the first and following versions of the manuscript dealing in particular with the methodological aspects of cost benefit analysis and substantially contributed to the final version. CL carried out first data analyses and decision trees. FG trained research assistants in rainy season. Supervised enrolment and data collection in the field in rainy season. Performed bibliographic research. Revised critically the manuscript. AA trained research assistants in dry season. Supervised enrolment and data collection in the field in dry season. Performed bibliographic research. Revised critically the manuscript. HT contributed to study design. Supervised enrolment and data collection in the field in both seasons. BN enrolled patients, trained and supervised research assistants, coordinated logistics in the field in both seasons. AR enrolled patients, trained and supervised research assistants, coordinated logistics in the field in both seasons. KVdE enrolled patients, trained and supervised research assistants, coordinated logistics in the field in the dry season. JVdE contributed with major inputs to the study design. Critically reviewed all draft versions, extensively collaborating to the final version. All authors read and approved the final version of the manuscript.

## References

[B1] Lubell Y Reyburn H Mbakilwa H Mwangi R Chonya S Whitty CJ Mills A The impact of response to the results of diagnostic tests for malaria: cost-benefit analysis BMJ 2008 336 202 205 10.1136/bmj.39395.696065.47 18199700 PMC2213875

[B2] Bualombai P Prajakwong S Aussawatheerakul N Congpoung K Sudathip S Thimasarn K Sirichaisinthop J Indaratna K Kidson C Srisuphanand M Determining cost-effectiveness and cost component of three malaria diagnostic models being used in remote non-microscope areas Southeast Asian J Trop Med Public Health 2003 34 322 333 12971557

[B3] Rolland E Checchi F Pinoges L Balkan S Guthmann JP Guerin PJ Operational response to malaria epidemics: are rapid diagnostic tests cost-effective? Trop Med Int Health 2006 11 398 408 10.1111/j.1365-3156.2006.01580.x 16553923

[B4] Shillcutt S Morel C Goodman C Coleman P Bell D Whitty CJ Mills A Cost-effectiveness of malaria diagnostic methods in sub-Saharan Africa in an era of combination therapy Bull World Health Organ 2008 86 101 110 10.2471/BLT.07.042259 18297164 PMC2647374

[B5] Yukich J D'Acremont V Kahama J Swai N Lengeler C Cost savings with rapid diagnostic tests for malaria in low-transmission areas: evidence from Dar es Salaam, Tanzania Am J Trop Med Hyg 2010 83 61 68 10.4269/ajtmh.2010.09-0632 20595479 PMC2912577

[B6] Chanda P Castillo-Riquelme M Masiye F Cost-effectiveness analysis of the available strategies for diagnosing malaria in outpatient clinics in Zambia Cost Eff Resour Alloc 2009 7 5 10.1186/1478-7547-7-5 19356225 PMC2676244

[B7] Uzochukwu BS Obikeze EN Onwujekwe OE Onoka CA Griffiths UK Cost-effectiveness analysis of rapid diagnostic test, microscopy and syndromic approach in the diagnosis of malaria in Nigeria: implications for scaling-up deployment of ACT Malar J 2009 8 265 10.1186/1475-2875-8-265 19930666 PMC2787522

[B8] Mosha JF Conteh L Tediosi F Gesase S Bruce J Chandramohan D Gosling R Cost implications of improving malaria diagnosis: findings from north-eastern Tanzania PLoS One 2010 5 e8707 10.1371/journal.pone.0008707 20090933 PMC2806838

[B9] Zurovac D Njogu J Akhwale W Hamer DH Larson BA Snow RW Effects of revised diagnostic recommendations on malaria treatment practices across age groups in Kenya Trop Med Int Health 2008 13 784 787 10.1111/j.1365-3156.2008.02072.x 18482078 PMC2592476

[B10] WHO Guidelines for the treatment of malaria 2010 second Geneva: WHO

[B11] WHO Guidelines for the treatment of malaria 2006 Geneva: WHO 2006

[B12] WHO Malaria Rapid Diagnostic Test Performance round 1. Results of WHO product testing of malaria RDTs (2009) 2010 Geneva: WHO

[B13] WHO Malaria Rapid Diagnostic Test Performance round 2. Results of WHO product testing of malaria RDTs (2009) 2010 Geneva: WHO

[B14] Bisoffi Z Sirima SB Menten J Pattaro C Angheben A Gobbi F Tinto H Lodesani C Neya B Gobbo M Van den Ende J Accuracy of a rapid diagnostic test on the diagnosis of malaria infection and of malaria-attributable fever during low and high transmission season in Burkina Faso Malar J 2010 9 192 10.1186/1475-2875-9-192 20609211 PMC2914059

[B15] Bisoffi Z Sirima BS Angheben A Lodesani C Gobbi F Tinto H Van den Ende J Rapid malaria diagnostic tests vs. clinical management of malaria in rural Burkina Faso: safety and effect on clinical decisions. A randomized trial Trop Med Int Health 2009 14 491 498 10.1111/j.1365-3156.2009.02246.x 19222821

[B16] Greenberg AE Ntumbanzondo M Ntula N Mawa L Howell J Davachi F Hospital-based surveillance of malaria-related paediatric morbidity and mortality in Kinshasa, Zaire Bull World Health Organ 1989 67 189 196 2743538 PMC2491235

[B17] Rafael ME Taylor T Magill A Lim YW Girosi F Allan R Reducing the burden of childhood malaria in Africa: the role of improved diagnostics Nature 2006 444 Suppl 1 39 48 17159893 10.1038/nature05445

[B18] Msellem MI Martensson A Rotllant G Bhattarai A Stromberg J Kahigwa E Garcia M Petzold M Olumese P Ali A Björkman A Influence of rapid malaria diagnostic tests on treatment and health outcome in fever patients, Zanzibar: a crossover validation study PLoS Med 2009 6 e1000070 10.1371/journal.pmed.1000070 19399156 PMC2667629

[B19] D'Acremont V Malila A Swai N Tillya R Kahama-Maro J Lengeler C Genton B Withholding antimalarials in febrile children who have a negative result for a rapid diagnostic test Clin Infect Dis 2010 51 506 511 10.1086/655688 20642354

[B20] Ansah EK Narh-Bana S Epokor M Akanpigbiam S Quartey AA Gyapong J Whitty CJ Rapid testing for malaria in settings where microscopy is available and peripheral clinics where only presumptive treatment is available: a randomised controlled trial in Ghana BMJ 2010 340 c930 10.1136/bmj.c930 20207689 PMC2833239

[B21] Njama-Meya D Clark TD Nzarubara B Staedke S Kamya MR Dorsey G Treatment of malaria restricted to laboratory-confirmed cases: a prospective cohort study in Ugandan children Malar J 2007 6 7 10.1186/1475-2875-6-7 17239256 PMC1797179

[B22] Williams HA Causer L Metta E Malila A O'Reilly T Abdulla S Kachur SP Bloland PB Dispensary level pilot implementation of rapid diagnostic tests: an evaluation of RDT acceptance and usage by providers and patients--Tanzania, 2005 Malar J 2008 7 239 10.1186/1475-2875-7-239 19019233 PMC2613413

[B23] Hamer DH Ndhlovu M Zurovac D Fox M Yeboah-Antwi K Chanda P Sipilinyambe N Simon JL Snow RW Improved diagnostic testing and malaria treatment practices in Zambia JAMA 2007 297 2227 2231 10.1001/jama.297.20.2227 17519412 PMC2674546

[B24] Fox-Rushby JA Hanson K Calculating and presenting disability adjusted life years (DALYs) in cost-effectiveness analysis Health Policy Plan 2001 16 326 331 10.1093/heapol/16.3.326 11527874

[B25] United Nations World Population Prospects The 2008 Revision. United Nations 2009 2010

[B26] IDA foudation Web catalogue 2010 http://www.idafoundation.org/we-offer/web-catalogue.html

[B27] Bisoffi Z Van den Ende J Costs of treating malaria according to test results BMJ 2008 336 168 169 10.1136/bmj.39401.486655.80 18199699 PMC2213792

[B28] Skarbinski J Ouma PO Causer LM Kariuki SK Barnwell JW Alaii JA de Oliveira AM Zurovac D Larson BA Snow RW Rowe AK Laserson KF Akhwale WS Slutsker L Hamel MJ Effect of malaria rapid diagnostic tests on the management of uncomplicated malaria with artemether-lumefantrine in Kenya: a cluster randomized trial Am J Trop Med Hyg 2009 80 919 926 19478249

[B29] Reyburn H Mbakilwa H Mwangi R Mwerinde O Olomi R Drakeley C Whitty CJ Rapid diagnostic tests compared with malaria microscopy for guiding outpatient treatment of febrile illness in Tanzania: randomised trial BMJ 2007 334 403 10.1136/bmj.39073.496829.AE 17259188 PMC1804187

[B30] Bjorkman A Martensson A Risks and benefits of targeted malaria treatment based on rapid diagnostic test results Clin Infect Dis 2010 51 512 514 10.1086/655689 20642355

